# Simple Low-Cost Production of DNA MS2 Virus-Like Particles As Molecular Diagnostic Controls

**DOI:** 10.1089/genbio.2022.0033

**Published:** 2022-12-21

**Authors:** Michael A. Crone, Paul S. Freemont

**Affiliations:** ^1^ London Biofoundry, Imperial College Translation and Innovation Hub, London, United Kingdom; Imperial College London, London, United Kingdom.; ^2^ Section of Structural and Synthetic Biology, Department of Infectious Disease, Imperial College London, London, United Kingdom; and Imperial College London, London, United Kingdom.; ^3^ UK Dementia Research Institute Care Research and Technology Centre, Imperial College London, London, United Kingdom.

## Abstract

Suitable controls are integral for the validation and continued quality assurance of diagnostic workflows. Plasmids, DNA, or *in vitro* transcribed RNA are often used to validate novel diagnostic workflows, however, they are poorly representative of clinical samples. RNA phage virus-like particles (VLPs) packaged with exogenous RNA have been used in clinical diagnostics as workflow controls, serving as surrogates for infectious viral particles. Comparable controls for DNA viruses are more challenging to produce, with analogous DNA phages being infectious and packaging of DNA within RNA phages requiring complex purification procedures and expensive chemical linkers. We present a simple and inexpensive method to produce *Emesvirus zinderi* (MS2) VLPs, packaged with DNA, that makes use of affinity chromatography for purification and enzymatic production of exogenous DNA suitable for packaging. The produced VLPs were packaged with hepatitis B virus DNA and were then quantified using droplet digital PCR and calibrated against the WHO international standard using a commercial assay in an accredited clinical laboratory.

## Introduction

The development of novel nucleic acid diagnostic assays for viral diseases relies on the availability of suitable materials for validation and continued quality assurance. The assessment of specificity, sensitivity, accuracy, precision, and, particularly, limit of detection requires materials that are accurately quantified and representative of the infectious agent. When available, World Health Organization international standards serve as the primary reference material^1^ and are often produced using clinical samples or cell culture-derived viral particles.

However, due to delays in availability, safety concerns,^2^ or limited access, there is often a lag where a diagnostic assay is urgently needed and no traceable metrological standards are available. To fill this gap, interim working standards are often produced by laboratories or companies to enable proficiency testing and expedite assay development^3^ and are later calibrated against the international standard.

Working standards can be derived from a variety of different materials. For RNA viruses, *in vitro* transcribed RNA,^2,4^ heat-inactivated virus particles (sold by Zeptometrix and Qnostics), or other viral particles packaged with exogenous RNA^5,6^ have all been utilized as working standards. For DNA viruses, plasmids,^7^ PCR products,^8^ and viral particles packaged with exogenous DNA^9–11^ have been utilized.

Although unprotected nucleic acids are popular as a part of the initial validation of diagnostic assays, alternative controls are often recommended when performing assay validation and proficiency testing within clinical laboratories.^12,13^ This is because unprotected nucleic acids are degraded by nucleases when spiked into sample matrices (such as plasma or sputum) and do not serve as processing controls as they do not require extraction.^9^

Within diagnostic laboratories, the use of phages as validation and proficiency controls has become increasingly popular.^12^ The *Emesvirus zinderi* (MS2) bacteriophage, a nonenveloped virus, is widely accepted as an RNA virus surrogate and has been described for veterinary applications^14^ and as a surrogate for influenza A,^15^ SARS-CoV,^15^ HIV,^16^ and SARS-CoV-2.^17,18^ Optimized affinity chromatography protocols have also been described, simplifying production and enabling preparation without expensive equipment.^19^ Analogous DNA phages have been suggested for the preparation of “armored DNA,” but these phages can infect other bacteria,^9^ suffer from low packaging efficiency,^20^ are complicated to produce,^11^ or are less stable in sample matrices than their RNA counterparts.^13^

In an attempt to create more stable surrogates for DNA viral particles, the MS2 RNA phage system has been successfully adapted for the packaging of exogenous DNA,^21^ but the process is expensive and complex, making it unlikely to be widely adopted. In this study, we describe a simplified protocol to produce MS2 virus-like particles (VLPs), making use of affinity chromatography and enzymatic digestion of exogenous DNA, to enable the inexpensive manufacturing of DNA-encapsulated particles. We characterize the produced particles using dynamic light scattering (DLS), quantitative PCR (qPCR), droplet digital PCR (ddPCR), and, finally, validate them using a commercial hepatitis B virus (HBV) assay within an accredited clinical laboratory.

## Results

The MS2 bacteriophage does not natively package DNA and, therefore, an adapted protocol is required to package exogenous DNA sequences. Nucleic acids require a structured sequence-specific segment known as a *pac* site^5,21–23^ to be spontaneously packaged within disassembled MS2 phages. Zhang et al have previously shown that this single-stranded portion, called a translational operator DNA (TR-DNA), can be conjugated to a PCR product using chemically modified primers,^22^ but their approach is complicated and expensive (Supplementary Table S1). We attempted to find a suitable enzymatic replacement to achieve the same goal, exogenous DNA that encompasses a double-stranded DNA product, and a single-stranded 5′ *pac* site.

**FIG. 1.**
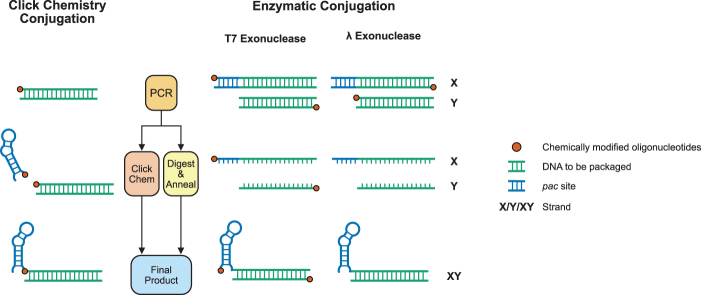
Comparison of the click chemistry technique used in Zhang et al^22^ with the T7 exonuclease and λ exonuclease enzymatic methods. X and Y are used to denote the strand with the 5′ TR-DNA extension (X) and the shorter strand without it (Y). For the click chemistry approach sulfhydryl-modified primers are used to perform the initial PCR and these are conjugated to an oligonucleotide containing the *pac* site that has an amine modification.^22^ For T7 exonuclease digestion, primers are chemically modified by the addition of phosphorothioate bonds to the first five bases of the protected oligonucleotide. The addition of these bonds protects the strands from digestion and the final product, therefore, incorporates the protected oligonucleotides (positive selection). In contrast, for λ exonuclease digestion, the primers are chemically modified by phosphorylation. λ exonuclease then preferentially digests the strands that have been phosphorylated, leaving a final product that does not incorporate the phosphorylated oligonucleotides (negative selection). TR-DNA, translational operator DNA.

It has been previously reported that there are multiple enzymatic methods for the production of single-stranded DNA.^24^ We explored the use of exonuclease and T7 exonuclease to generate single-stranded DNA with the goal that two single-stranded DNA strands (complementary to one another, other than a short 5′ extension containing the *pac* site) could then be annealed successfully to generate the required DNA product. The two enzymes contrast in their digestion mechanisms, with λ exonuclease preferentially digesting phosphorylated strands, a form of negative selection, and T7 exonuclease preferentially digesting unprotected strands (strands that have not been phosphorothioated), a form of positive selection (Fig. 1).

We benchmarked λ exonuclease and T7 exonuclease using two criteria: (1) the extent to which the double-stranded product is digested to its single-stranded form and (2) the total yield of the final annealed product of the digested X and Y products. We showed that although it was reported that λ exonuclease did not have activity when directly added to an unpurified PCR product,^24^ the enzyme did in fact digest a double-stranded PCR product to a single-stranded product (Supplementary Fig. S1).

However, as reported previously,^24^ phosphorylated oligonucleotides ordered from DNA synthesis companies are not all successfully phosphorylated, leading to only partially digested products. Since our yield of final exogenous DNA product relies on near or total digestion of the generated PCR product to its single-stranded form, λ exonuclease does not perform well using our benchmark criteria.

**FIG. 2.**
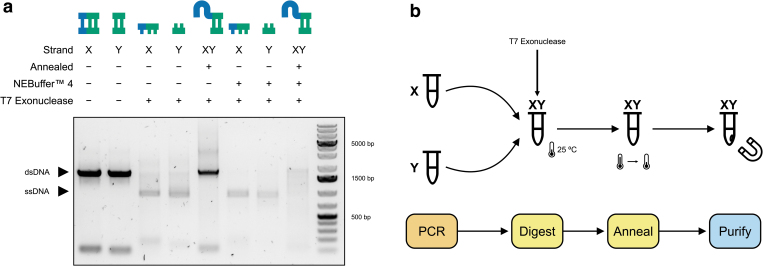
Preparation of DNA suitable for packaging inside MS2 VLPs. **(a)** Agarose electrophoresis of optimization with overnight T7 exonuclease digestion. The protected strand (X or Y or, in the case of annealed, XY) is indicated together with whether the product was annealed and the presence of reaction buffer and T7 exonuclease. T7 exonuclease showed activity both with and without addition of its reaction buffer. X and Y strands could also be combined, digested, and subsequently annealed (XY) in a single-pot format. **(b)** The final optimized procedure for producing exogenous DNA for packaging. PCRs with protected X and Y strands are performed separately and then combined and supplemented with T7 exonuclease that digests the unprotected strands overnight before a final annealing step in a thermocycler. The annealed product is then purified using magnetic beads. The strand present in each tube (X, Y, or XY) is shown at each step.

In contrast, since T7 exonuclease digests all unprotected DNA (positive selection), there is no requirement for all oligonucleotides to have the required phosphorothioate bonds to ensure complete digestion to a single-stranded product as all oligonucleotides that do not have the required bonds will be digested anyway. We show that T7 exonuclease displays activity when added directly to unpurified PCR products both with and without addition of its requisite reaction buffer (Fig. 2a).

We further demonstrated that X and Y PCRs can be combined with T7 exonuclease, digested, and subsequently annealed successfully in a one-pot format (workflow Fig. 2b). The addition of T7 exonuclease to the X and Y PCRs without its accompanying reaction buffer and incubated overnight (Supplementary Fig. S2; Fig. 2a) showed the best performance in terms of our benchmark criteria and, therefore, these optimised reaction conditions were taken forward.

**FIG. 3.**
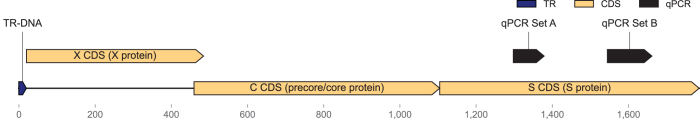
Diagram of the amplified DNA product, containing the TR-DNA and three HBV genes. The assay targets of the duplex qPCR assay (qPCR sets A and B) are also displayed. Diagram was generated using DNA Features Viewer.^35^ HBV, hepatitis B virus; qPCR, quantitative PCR.

Using our optimized approach (Fig. 2b), we amplified a portion of the HBV containing the *X*, *C*, and *S* genes (Fig. 3), which are targeted by several published^25,26^ and commercial assays. We then prepared the amplified product for packaging by digesting, annealing, and subsequently purifying the products using magnetic beads to remove any contaminating oligonucleotides and primer dimers (Supplementary Fig. S3).

**FIG. 4.**
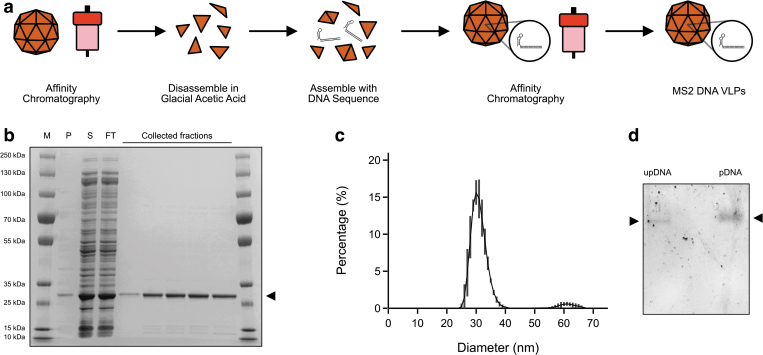
Preparation and biochemical characterisation of DNA MS2 VLPs. **(a)** Preparation of DNA VLPs by protein purification of empty particles, followed by disassembly, assembly with exogenous DNA, and finally a second affinity chromatography step to isolate packaged DNA VLPs. **(b)** SDS-PAGE gel electrophoresis of the initial affinity chromatography includes a protein marker (M) followed by pellet (P) and soluble fraction (S) of the cell lysate, followed by the column FT and collected elution fractions, where the coat protein dimer (∼28 kDa) is indicated by an arrow. **(c)** Dynamic light scattering analyses of the MS2 DNA VLPs, showing a predominantly monodisperse population of ∼30 nm. Error bars represent the SD of three technical replicates. **(d)** Gel shift assay showing the upDNA and pDNA within the VLP run side by side. pDNA, packaged DNA; SD, standard deviation; SDS-PAGE, sodium dodecyl sulfate-polyacrylamide gel electrophoresis; upDNA, unpackaged DNA; VLP, virus-like particle.

We proceeded to produce the viral coat protein for packaging, disassembly, and subsequent reassembly (Fig. 4a). We first expressed and purified unpackaged MS2 VLPs using affinity chromatography (Fig. 4b) as previously described.^17^ The purified particles were disassembled using glacial acetic acid as performed by Wu et al^27^ and buffer exchanged out of glacial acetic acid and into a buffer compatible with downstream steps.

The disassembled coat protein dimers were then incubated together with the exogenous DNA and subsequently purified again using affinity chromatography. Reassembly was confirmed using DLS, showing a predominantly monodisperse peak at a diameter of ∼30 nm (Fig. 4c). Furthermore, packaging was confirmed by gel shift assay that showed a shift and more diffuse pattern of the packaged DNA when compared with the unpackaged DNA (Fig. 4d).

**FIG. 5.**
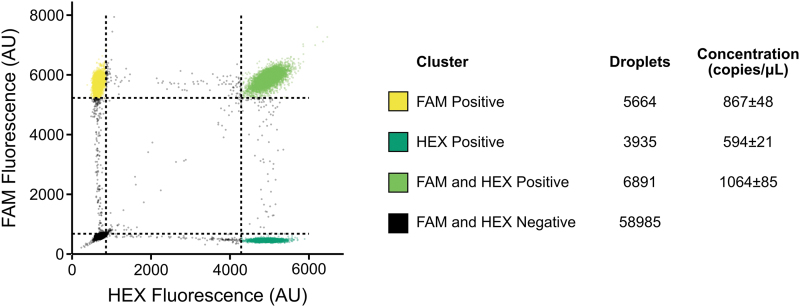
Droplet digital PCR (ddPCR) of prepared DNA MS2 VLPs. ddPCR of *n* = 3 aliquots of prepared VLPs with primer sets qPCR set A (FAM) and qPCR set B (HEX). Clustering was performed using K-Means on a single sample and the thresholds were extrapolated to the rest of the samples. Thresholds (dotted lines) are 3 SDs from the centroid of each cluster calculated using K-Means.

After our initial biochemical validation, we proceeded to validate the produced VLPs as molecular diagnostic controls. We first confirmed that the previously described duplex assay successfully amplified the two targets within our produced VLPs using qPCR (Supplementary Fig. S4). We also used this experiment to guide the production of aliquots within a suitable working range for further use. Then, to have an idea of the absolute concentration of the particles, we performed ddPCR on three individual aliquots (Fig. 5).

We used both primer and probe sets that we had previously validated with qPCR. Using two separate targets allowed for the determination of residual DNA found in the preparation of the VLPs. Any droplets positive for a single target were assumed to be residual DNA contamination that was not removed by DNase treatment. Concentrations are calculated using a Poisson distribution. At low concentrations, the number of droplets that contain duplicate strands of DNA can be considered negligible and, therefore, the clustering should be representative of droplets with either residual DNA or packaged VLPs.

We were able to calculate the relative concentration of residual DNA, which was ∼50% for qPCR set A and ∼30% for qPCR set B, and the concentration of our VLPs (∼50% for qPCR set A and ∼70% for qPCR set B). The majority of the qPCR signal is, therefore, generated by our VLPs. Total concentration was 1931 ± 133 copies per microliter for Set A and 1658 ± 106 copies per microliter for Set B.

**FIG. 6.**
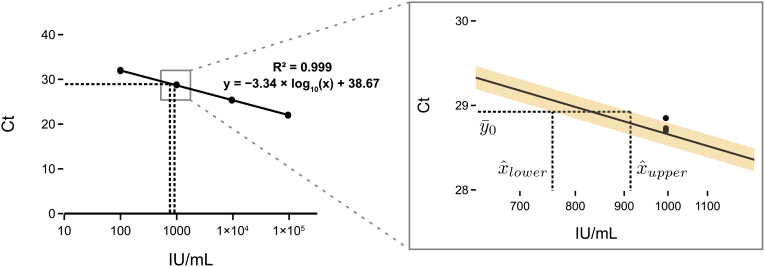
Calibration of produced VLP standard against the HBV international standard using a Roche cobas^®^ 6800 system and the Roche cobas HBV test. The standard curve was generated by serially diluting the 4th WHO international standard for HBV DNA for NAT. The standard curve was fit using linear regression (*n* = 3 technical replicates of each dilution). The PCR efficiency was 99.4% (Supplementary Methods). VLP aliquots were prepared by diluting our produced VLP in human plasma. The concentration of the VLP aliquots were then calculated using the fitted equation. Error is displayed as the 95% PI in yellow of *n* = 4 biological replicates using an adaptation of Fieller's Theorem.^33^ The inset shows the mean Ct value ($\bar{y_{0}}$) and the upper and lower bounds of the calculated VLP concentration ($\hat{x}$). PI, prediction interval.

Finally, we validated the generated particles by spiking them into human plasma and testing them using a commercial diagnostic qPCR assay for HBV. MS2 VLP preparations are stable when spiked into plasma^28^ and we showed consistent results over four biological replicates. Furthermore, we calibrated our developed standard against the 4th WHO international standard for HBV DNA for NAT using the commercial assay. Our working standard had a calibrated concentration of 832 IU/mL (95% prediction interval [PI] 757–914) (Fig. 6).

## Discussion

Here we describe the development of a novel approach for the generation of dsDNA with a single-stranded TR-DNA for packaging within MS2 VLPs. We combine this novel approach with affinity chromatography to demonstrate a simple low-cost method for the production of DNA MS2 VLPs. We then validate the use of these particles as viral diagnostic controls, using a previously described duplex assay, as well as a commercial assay within a clinical laboratory. As a part of this validation, we calibrate our developed standard against the 4th WHO international standard for HBV for nucleic acid testing (NAT).

Suitable controls for DNA viruses are not readily available, with many companies focused on providing either diluted infectious particles or, alternatively, unpackaged DNA in the form of plasmids or chemically synthesized DNA. Furthermore, these controls are expensive and international distribution is made complicated by shipping requirements for infectious samples.

For example, the WHO international standard for HBV DNA for NAT is formulated from plasma from an HBV carrier with a high viral load^29^ that has been diluted with HBV-negative plasma and subsequently aliquoted and assigned an international unit (IU) equivalent.^30^ The infectious nature of the standard means that it needs to be handled under very specific biosafety conditions and there are shipping restrictions that preclude its use in many settings.

We have previously shown how locally manufactured, robust, and reliable diagnostic standards can play a critical role in diagnostic workflow development.^17,18^ Our developed SARS-CoV-2 workflow is still making a significant contribution in diagnostic laboratories in London, with >1,500,000 tests performed by October 2022. Our previously developed SARS-CoV-2 VLPs have also been included in a Coronavirus Standards Harmonization Study as a part of the Coronavirus Standards Working Group. The only standard produced at an academic institution in the study, it performed robustly against its commercial counterparts.

Adequate pandemic preparedness and resilience within diagnostic laboratories requires ease of access to materials, not only for large commercial enterprises producing their own diagnostics for Emergency Use Authorization or conformité européenne marking, but also for diagnostic laboratories that are developing and maintaining their own in-house assays, also known as laboratory-developed tests, especially those in low- and middle-income countries (LMICs). The reliance on commercial standards alone creates inequity in the development of clinical diagnostics.

Empowering regional centers to manufacture their own standards would provide a means to democratize diagnostic assay development. By leveraging the OpenMTA,^31^ academic institutions can provide the necessary materials and expertise, facilitating technology transfer and allowing for the decentralized production of biosafe “open source” standards, such as VLPs, that rely only on the availability of low-cost protein purification equipment.^17^

**The Bigger Picture**

**The poor availability of clinical samples for diagnostic development inspired us to develop accessible molecular diagnostic standards for both RNA and DNA viruses. Diagnostic standards for RNA and DNA viruses are sometimes commercially available, but there are inevitable delays when new standards for new diseases are needed, and they are often expensive with restrictions on shipping that precludes their use in many LMICs. Furthermore, the availability of commercial standards is dictated by the needs of laboratories in high-income countries.**

**Having already demonstrated that rapidly deployable RNA standards can play a pivotal role in diagnostic workflow development during a pandemic, we wanted to expand our work to include standards for DNA viruses. Our addition of the DNA VLP strengthens our open-source diagnostic standard toolkit. We foresee this toolkit empowering regional health agencies around the world to manufacture their own standards and, thereby, provide a means to democratize diagnostic assay development. Our hope is that this will be the first step in ending the dependence of diagnostic laboratories and health agencies in LMICs on large multinational diagnostics manufacturers.**

We believe that by providing a toolkit for the development of reliable, robust, and quantitative standards for RNA and DNA viruses and making it available under the OpenMTA, we can begin to enable laboratories around the world to develop their own diagnostic assays. Here we add the DNA MS2 VLP to this toolkit to complement our previously developed method for making low-cost RNA MS2 VLPs. We have made all required materials available under the OpenMTA to enable both commercial and academic institutions to work with the materials without limitations.

## Materials and Methods

### Primers and probes and plasmid to produce viral DNA sequence

Primers and probes were ordered from Integrated DNA Technologies and are given in Supplementary Tables S2 and S3. A construct containing the X, C, and S genes of the HBV (Accession No.: KY003230^32^) was ordered from GeneArt (ThermoFisher Scientific) and is available from Addgene (Addgene No. 179155).

### Production of partially single-stranded DNA for packaging using T7 exonuclease

Two sets of PCRs were set up to create the exogenous DNA for packaging. The X PCR was performed using a phosphorothiated forward primer and unprotected reverse primer and the Y PCR was performed with an unprotected forward primer and phosphorothiated reverse primer. PCRs were performed with a final primer concentration of 1 mM using Q5^®^ High-Fidelity 2X Master Mix (NEB). Equal volumes of X and Y PCRs were then combined, supplemented with 0.2 U/μL of T7 exonuclease (NEB) and incubated at 25°C overnight before being heated to 95°C and slowly annealed (−0.1°C/s) in a thermocycler.

Digested annealed exogenous DNA was concentrated in an Amicon^®^ Ultra 0.5 mL 30K Centrifugal Filter (Merck Millipore) and two washes were performed with Invitrogen TE Buffer (ThermoFisher Scientific). The final concentrated product was made up to 100 μL with Invitrogen TE Buffer (ThermoFisher Scientific), and 0.7 × AMPure XP beads (Beckman Coulter) were added and the product was purified according to the manufacturer's instructions (two washes with 80% ethanol were performed and the final product was eluted in UltraPure™ DNase/RNase-Free Distilled Water [ThermoFisher Scientific]). Eluted DNA was diluted and quantified using the Qubit™ dsDNA HS kit (ThermoFisher Scientific) on a Qubit 3 Fluorometer (ThermoFisher Scientific).

### Expression, purification, and disassembly of MS2 coat protein dimers and maturation protein

A plasmid construct containing only the maturation protein and his-tagged coat protein dimer (Addgene No. 179156) was transformed into Rosetta2™ (DE3) pLysS cells (Merck). An overnight culture was used to inoculate 200 mL of Terrific Broth (Merck) supplemented with 50 mg/mL of Kanamycin (Merck), and grown at 30°C, 200 rpm until an OD of 0.8. The culture was induced by supplementing with 0.5 mM IPTG (Merck) and grown at 30°C for a further 16 h. Cells were collected at 3220 *g* at 4°C and stored at −20°C for later purification.

All protein purification steps were performed at 4°C. The cell pellet was resuspended in 4 mL sonication buffer (50 mM Tris-HCl pH 8.0, 5 mM MgCl_2_, 5 mM CaCl_2_, and 100 mM NaCl) with 700 U RNase A (Qiagen), 2500 U BaseMuncher Endonuclease (Abcam), and 200 U TURBO DNase (ThermoFisher Scientific). The cells were sonicated for a total of 2 min (50% amplitude, 30 s on, 30 s off) on wet ice. The lysate was then incubated for 3 h at 37°C. The lysate was centrifuged at 10,000 *g* for 10 min at room temperature in a microcentrifuge. The supernatant was then filtered with a Minisart^®^ 5 μm cellulose acetate (SFCA) filter (Sartorius) before being mixed 1:1 with 2 × binding buffer (100 mM monosodium phosphate monohydrate pH 8.0, 30 mM imidazole, 600 mM NaCl).

Supernatant was applied to a 5 mL HiTrap^®^ TALON^®^ Crude column (Cytiva) on an ÄKTA pure (Cytiva) primed with binding buffer (50 mM monosodium phosphate monohydrate pH 8.0, 15 mM imidazole, 300 mM NaCl). The protein was eluted with elution buffer (50 mM monosodium phosphate monohydrate pH 8.0, 200 mM imidazole, 300 mM NaCl) and then desalted and buffer exchanged into STE buffer (10 mM Tris-HCl pH 7.5, 1 mM EDTA, 100 mM NaCl) using an Amicon Ultra-15 10K Centrifuge Filter (Merck Millipore). The protein concentration was measured using the Qubit Protein Assay Kit and Qubit 3 Fluorometer (Thermo Fisher Scientific).

The purified protein was incubated with cold glacial acetic acid (final concentration 66% v/v) for 30 min on ice before being centrifuged at 6600 *g* for 20 min at 4°C. The protein sample was then buffer exchanged into sonication buffer (50 mM Tris-HCl pH 8.0, 5 mM MgCl_2_, 5 mM CaCl_2_, and 100 mM NaCl) and concentrated down to 500 μL using an Amicon Ultra-15 10K Centrifuge Filter (Merck Millipore).

### Encapsulation of DNA within MS2 VLPs and final purification

Purified exogenous DNA was incubated in a Protein LoBind tube (Eppendorf) with a 10-fold molar excess of disassembled MS2 coat protein dimers for 3 h at room temperature before being incubated for 36 h at 4°C. Sonication buffer was added up to 1.5 mL, supplemented with 100 U of TURBO DNase (ThermoFisher Scientific) and incubated for 90 min at 37°C.

The sample was then mixed 1:1 with 2 × binding buffer (100 mM monosodium phosphate monohydrate pH 8.0, 30 mM imidazole, 600 mM NaCl) and was applied to a 5 mL HiTrap TALON Crude column (Cytiva) on an ÄKTA pure (Cytiva) primed with binding buffer (50 mM monosodium phosphate monohydrate pH 8.0, 15 mM imidazole, 300 mM NaCl). The protein was eluted with elution buffer (50 mM monosodium phosphate monohydrate pH 8.0, 200 mM imidazole, 300 mM NaCl) and then desalted and buffer exchanged into STE buffer (10 mM Tris-HCl pH 7.5, 1 mM EDTA, 100 mM NaCl) using an Amicon Ultra-15 30K Centrifuge Filter (Merck Millipore). All DNA MS2 aliquots were stored at −80°C.

### Dynamic light scattering

DLS was performed using a Zetasizer Nano (Malvern Panalytical) according to the manufacturer's instructions.

### Gel shift assay

The gel shift assay was run as a 1% agarose gel and poststained with SYBR Gold nucleic acid stain (ThermoFisher Scientific).

### Quantitative PCR

qPCR was performed using a previously described duplex assay^25^ with the primers and probes given in Supplementary Table S3. VLPs were lysed by heating to 95°C for 5 min in a thermocycler before being added to qPCRs. qPCRs (20 μL) used TaqPath™ qPCR Master Mix, CG (ThermoFisher Scientific) with final primer and probe concentrations of 400 and 200 nM, and a sample volume of 1 μL. The reaction mixture was then thermocycled (50°C for 2:00, 95°C for 5:00 and 45 cycles of 95°C for 0:10, and 60°C for 1:00) on a BioRad CFX96 qPCR machine.

### Droplet digital PCR

ddPCR was performed using a previously described duplex assay^25^ with the primers and probes given in Supplementary Table S3. VLPs were lysed by heating to 95°C for 5 min in a thermocycler before being added to ddPCRs. ddPCRs (20 μL) used ddPCR™ Multiplex Supermix (Bio Rad) with final primer and probe concentrations of 900 and 250 nM and a sample volume of 2 μL. Droplets were then generated using a QX200™ Droplet Generator (Bio Rad) and transferred to a PCR plate and sealed, all according to the manufacturer's instructions.

The reaction mixture was then thermocycled (95°C for 10:00, 40 cycles of 94°C for 0:30, and 60°C for 1:00, and a final enzyme inactivation step of 98°C for 10:00) on a BioRad C1000 touch PCR machine. Finally, droplets were read using the QX200 Droplet Reader (Bio Rad). Data were then analyzed using a Python implementation (https://github.com/mcrone/plotlydefinerain) of an online tool called Defining The Rain (https://definetherain.org.uk/) with added support for two color channels.

### Calibration against WHO international standard using a commercial assay

The 4th WHO international standard for HBV DNA for NAT was ordered from NIBSC. The standard was resuspended according to the instructions for use. Calibration curve samples were generated by diluting the international standard with human plasma (Merck) to create a standard curve ranging from 955,000 to 95.5 IU/mL with three technical replicates of each concentration. Aliquots of our working standard were generated by spiking 50 μL of our generated VLPs into 950 μL of human plasma (Merck). The calibration curve samples and the aliquots of working standard were then tested using a cobas^®^ 6800 system (Roche) and cobas HBV test (Roche) according to the manufacturer's instructions.

The working standard calibration was performed by fitting of the standard curve using linear regression, and the error was calculated using a statistical tool^33^ based on Fieller's theorem.^34^ Further details are available in the Supplementary Methods. Residuals of the linear regression are shown in Supplementary Figure S5. A Python implementation of the calculation of the PIs and plots is available on GitHub (https://github.com/mcrone/qpcr_abs_calibration). All results are available in Supplementary Table S4.

## Supplementary Material

Supplemental data

Supplemental data

Supplemental data

Supplemental data

Supplemental data
