# Supplementary material for: Simple Low-Cost Production of DNA MS2 Virus-Like Particles As Molecular Diagnostic Controls
Source: GEN Biotechnol. 2022 Dec 21;1(6):496–503. doi: 10.1089/genbio.2022.0033 (PMC9814128; doi:10.1089/genbio.2022.0033)
Supplement: Supplemental data [file Supp_TableS1.pdf]

Supplementary Tables

Supplementary Table 1. Cost comparison between the method described by Zhang et al. and the method described in this study for the production of a single DNA VLP. Costs of common consumables like pipette tips and chemicals used to make buffers have been omitted. Pricing is current as of October 2022, but could be subject to change.

					Zhang et al.[2]	This Study
**Stage**	**Item**	**Manufacturer**	**Catalog Number**	**List Price**	**Per VLP**	**Per VLP**
Exogenous DNA Production	DNA Synthesis (1000 bp)	Twist Bioscience		£50	£50	£50
	Oligo Synthesis	IDT			£100	£45
	Q5® High-Fidelity 2X Master Mix	NEB	M0492S	£134	£21	£43
	Amicon® Ultra 0.5mL 3kDa	Merck Millipore	UFC500308	£49.60	£6.20	
	Amicon® Ultra 0.5mL 50kDa	Merck Millipore	UFC505008	£49.60	£12.40	
	Zeba™ Spin Desalting Columns, 40K MWCO, 0.5 mL	ThermoFisher Scientific	87766	£154.00	£6.16	
	T7 Exonuclease	NEB	M0263S	£57		£18
	AMPure XP	Beckman Coulter	A63880	£230.90		£3.23
	Amicon® Ultra 0.5mL 30kDa	Merck Millipore	UFC503008	£49.60		£6.20
	TE Buffer	ThermoFisher Scientific	12090015	£47	£0.47	£0.47
				Total DNA production	£196.67	£166.02
Protein Purification	TURBO™ DNase	ThermoFisher Scientific	AM2238	£125	£18.75	£18.75
	RNase A	Qiagen	19101	£225	£9.00	£9.00
	Basemuncher	Abcam	ab270049	£155	£15.50	£15.50
	HiPrep 16/60 Sephacryl S-200 HR	Cytiva	17116601	£676	£676	
	HiLoad 16/600 Superdex 75 pg	Cytiva	28989333	£2,198	£2,198	
	SnakeSkin™ Dialysis Tubing, 10K MWCO, 22 mm	ThermoFisher Scientific	68100	£184	£5	
	Minisart® Syringe Filter, SFCA, Pore Size 5 mm	Sartorius	17594	£848		£1.70
	HiTrap TALON crude	Cytiva	28953767	£636		£127
	Amicon® Ultra-15 10 kDa (2)	Merck Millipore	UFC901008	£104.00		£26.00
	Amicon® Ultra-15 30 kDa	Merck Millipore	UFC903008	£104.00		£13.00
				Total Protein Purification	£2,921.85	£211.15
				**Total Cost**	**£3,118.52**	**£377.17**
