# Supplementary material for: Simple Low-Cost Production of DNA MS2 Virus-Like Particles As Molecular Diagnostic Controls
Source: GEN Biotechnol. 2022 Dec 21;1(6):496–503. doi: 10.1089/genbio.2022.0033 (PMC9814128; doi:10.1089/genbio.2022.0033)
Supplement: Supplemental data [file Supp_FigS1-S3.pdf]

Supplementary Figures

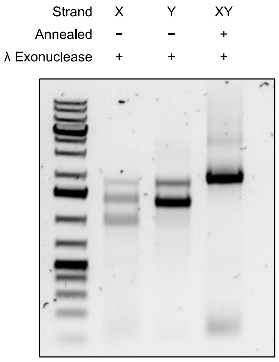

Supplementary Figure 1. Agarose Electrophoresis showing digestion with λ Exonuclease. λ Exonuclease was added directly to the completed PCR reactions without reaction buffer.

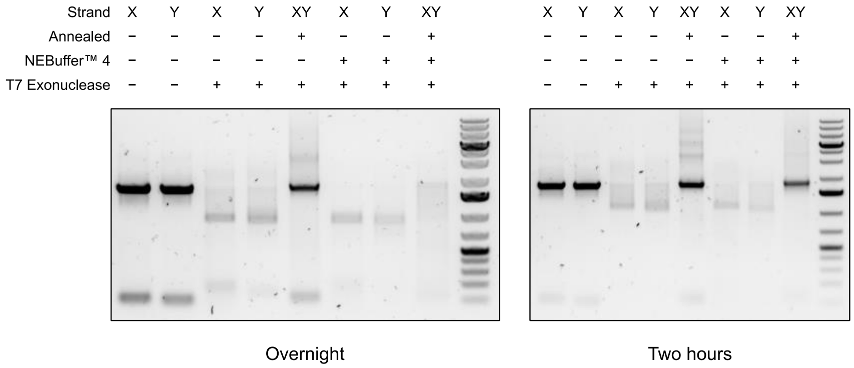

Supplementary Figure 2. Agarose Electrophoresis showing optimisation of digestion conditions with T7 Exonuclease. Reactions were tested with and without buffer, individually and after adding F and R together and with a two-hour digestion or overnight at 25 °C.

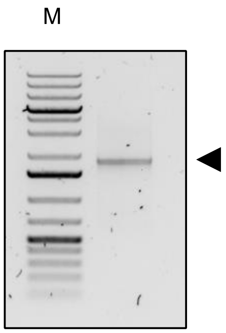

Supplementary Figure 3. Agarose Electrophoresis with DNA Ladder (M) and magnetic bead purified product after digestion and annealing (indicated with the arrow).
