# Supplementary material for: Simple Low-Cost Production of DNA MS2 Virus-Like Particles As Molecular Diagnostic Controls
Source: GEN Biotechnol. 2022 Dec 21;1(6):496–503. doi: 10.1089/genbio.2022.0033 (PMC9814128; doi:10.1089/genbio.2022.0033)
Supplement: Supplemental data [file Supp_FigS4-S5.pdf]

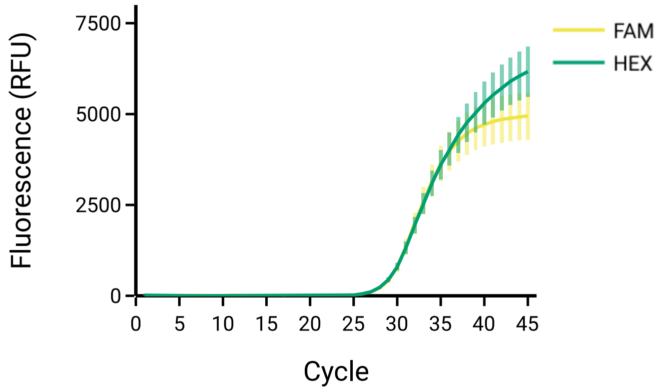

Supplementary Figure 4. Raw qPCR curves of DNA MS2 VLPs detected with the duplex assay.[1] Error bars represent the SD of n = 5 technical replicates.

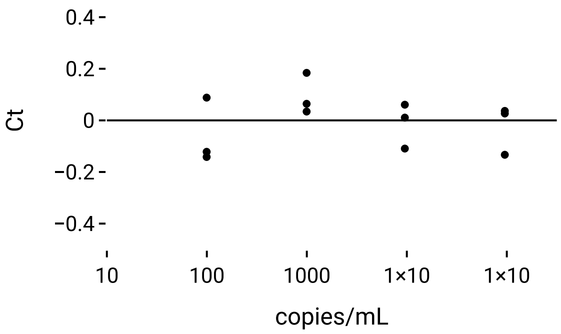

Supplementary Figure 5. Residuals obtained after fitting the standard curve using linear regression (SEq 1).
