# Supplementary material for: Simple Low-Cost Production of DNA MS2 Virus-Like Particles As Molecular Diagnostic Controls
Source: GEN Biotechnol. 2022 Dec 21;1(6):496–503. doi: 10.1089/genbio.2022.0033 (PMC9814128; doi:10.1089/genbio.2022.0033)
Supplement: Supplemental data [file Supp_TableS2-S4.pdf]

Supplementary Table 2. Oligonucleotides required for exogenous DNA amplification

Process	Forward Sequence	Reverse Sequence
Exogenous DNA [T7 Exonuclease]	A*A*C*A*T*GAGGATTACCCATGTATGGCTGCTAGGCTGTACTGC	TTAAATGTATACCCAGAGACAAAAGAAAATTG
	ATGGCTGCTAGGCTGTACTGC	T*T*A*A*A*TGTATACCCAGAGACAAAAGAAAATTG
Exogenous DNA [Lambda Exonuclease]	AACATGAGGATTACCCATGTATGGCTGCTAGGCTGTACTGC	/5Phos/TTAAATGTATACCCAGAGACAAAAGAAAATTG
	/5Phos/ATGGCTGCTAGGCTGTACTGC	TTAAATGTATACCCAGAGACAAAAGAAAATTG

Supplementary Table 3. Oligonucleotides required for qPCR and ddPCR quantification (derived from a previously published assay[1]).

Set	Forward Sequence	Reverse Sequence	Probe Sequence
A	GTCCTCCAATTTGTCCTGG	TGAGGCATAGCAGCAGGAT	/56-FAM/CTGGATGTG/ZEN/TCTGCGGCGTTTTATCAT/3IABkFQ/
B	CACCTGTATTCCCATCCCATC	AGCCCTACGAACCACTGAACA	/5HEX/AAACGGACT/ZEN/GAGGCCCACTCCCA/3IABkFQ/

Supplementary Table 4. Raw Roche® cobas 6800 results for the diluted International Standard and VLP samples using the Roche cobas® HBV assay.

Sample	Target 1 Ct	Measured IU/mL	QS Ct	QS Result	Valid?
95500	21.92	107000	32.43	Valid	Yes
95500	22.08	98200	32.46	Valid	Yes
95500	22.09	85100	32.26	Valid	Yes
9550	25.28	15100	32.93	Valid	Yes
9550	25.45	8390	32.24	Valid	Yes
9550	25.4	9140	32.32	Valid	Yes
995	28.73	940	32.33	Valid	Yes
995	28.7	1010	32.41	Valid	Yes
995	28.85	1030	32.58	Valid	Yes
99.5	32.09	98	32.4	Valid	Yes
99.5	31.86	115	32.4	Valid	Yes
99.5	31.88	147	32.77	Valid	Yes
VLP	28.81	932	32.4	Valid	Yes
VLP	28.8	835	32.23	Valid	Yes
VLP	29.13	908	32.68	Valid	Yes
VLP	28.96	756	32.25	Valid	Yes
Blank	-	Target Not Detected	32.41	Valid	Yes
