# Supplementary material for: Simple Low-Cost Production of DNA MS2 Virus-Like Particles As Molecular Diagnostic Controls
Source: GEN Biotechnol. 2022 Dec 21;1(6):496–503. doi: 10.1089/genbio.2022.0033 (PMC9814128; doi:10.1089/genbio.2022.0033)
Supplement: Supplemental data [file Supp_Data.pdf]

Supplementary Methods

Production of partially single stranded DNA for packaging using λ Exonuclease

Two sets of PCR reactions were setup to create the exogenous DNA for packaging. The X PCR was performed using a phosphorylated reverse primer and unmodified forward primer and the Y PCR was performed with a phosphorylated forward primer and unmodified reverse primer. PCR reactions were performed with a final primer concentration of 1 µM using Q5® High-Fidelity 2X Master Mix (NEB). Equal volumes of X and Y PCR reactions were then combined, supplemented with 0.2 U/µL of λ Exonuclease (ThermoFisher Scientific) and incubated at 37 °C for 30 minutes before being heated to 95 °C and slowly annealed (-0.1 °C/sec) in a thermocycler.

Concentration and error calculation for working standard calibration

The working standard calibration was performed using linear regression and the error was calculated using a stastistical tool[3] based on Fieller's theorem.[4] For completeness the equations below are included as found in Pizzamiglio et al.[3]

The data from the standard curve was initially fit by linear regression to SEq 1. The residuals obtained using the fitted equation can be visualized in Supplementary Figure 5.

$$y_{ij} = \beta_0 + \beta_1 x_i$$ **SEq 1**

where y_{ij} specifies the value of the Ct values determined for the j-th replicate (j = 1,2,...,Ji) at log x_i different (i = 1,2,...,I) WHO international standard dilutions.

PCR efficiency could then be calculated using SEq 2:

$$\text{Efficiency} = 10^{(-1/\beta_1)} - 1$$ **SEq 2**

The estimate of the unknown log starting concentration ($\hat{x}_0$) of the unknown VLP concentration (x_0) is then calculated by substituting the mean Ct of the replicates ($\bar{y}$) of the VLP working standard into SEq 3.

$$\hat{x}_0 = \frac{\bar{y}-\beta_0}{\beta_1}$$ **SEq 3**

The variance for the standard was then estimated using SEq 4.

$$s_p^2 = \frac{\sum_{i=1}^{I}\sum_{j=1}^{J_i}(y_{ij}-\bar{y}_i)^2}{\sum_{i=1}^{I}(J_i-1)}$$ **SEq 4**

where $\bar{y}_i$ is the mean of the Ct values at the i-th standard dilution

We can then define the deviance (s_{xx}) of the x_i values as SEq 5:

$$s_{xx} = \sum_{i=1}^{I} J_i(x_i - \bar{\bar{x}})^2$$ **SEq 5**

And the mean ($\bar{\bar{x}}$) of the x_i values as SEq 6:

$$\bar{\bar{x}} = \frac{\sum_{i=1}^{I} J_i x_i}{\sum_{i=1}^{I} J_i} \qquad \textbf{SEq 6}$$

$t_{f;1-\alpha/2}$ is the value which corresponds to a critical value where the significance is α and there are f degrees of freedom.

The confidence intervals of x_0 are obtained by calculating the roots of the quadratic equation SEq 7.

$$Ax^2 + 2Bx + C = 0 \qquad \textbf{SEq 7}$$

where:

$$A = \beta_1^2 - \frac{s_p^2}{s_{xx}} t_{f;1-\alpha/2}^2 \qquad \textbf{SEq 8}$$

$$B = \beta_0\beta_1 - \bar{y}\beta_1 + \frac{s_p^2 \bar{\bar{x}}}{s_{xx}} t_{f;1-\alpha/2}^2 \qquad \textbf{SEq 9}$$

$$C = \bar{y}^2 + \beta_0^2 - 2\bar{y}\beta_0 - \frac{s_p^2}{K} t_{f;1-\alpha/2}^2 - \left(\frac{s_p^2 \sum_{i=1}^{I} J_i x_i^2}{s_{xx} \sum_{i=1}^{I} J_i} \right) t_{f;1-\alpha/2}^2 \qquad \textbf{SEq 10}$$

finally, by defining g as:

$$g = \frac{s_p^2 t_{f;1-\alpha/2}^2}{s_{xx}\beta_1^2} \qquad \textbf{SEq 11}$$

We can obtain the two roots of SEq 7 and the required confidence limits of x_0 using SEq 12:

$$\left.\begin{matrix} \hat{x}_{upper} \\ \hat{x}_{lower} \end{matrix}\right\} = \hat{x}_0 + \frac{(\hat{x}_0 - \bar{\bar{x}})g \pm (s_p t_{f;1-\alpha/2}/\beta_1)\{[(\hat{x}_0 - \bar{\bar{x}})^2/s_{xx}] + (1-g)(1/K + 1/n)\}^{\frac{1}{2}}}{1-g} \qquad \textbf{SEq 12}$$

The prediction limits of the Ct value of the VLP sample ($\bar{y}$) are then calculated using SEq 13:

$$\left.\begin{matrix} \hat{y}_{upper} \\ \hat{y}_{lower} \end{matrix}\right\} = \bar{y} \pm t_{f;1-\alpha/2} s_p \left(\frac{(x - \bar{\bar{x}})^2}{s_{xx}} + \frac{1}{K} + \frac{1}{n} \right)^{\frac{1}{2}} \qquad \textbf{SEq 13}$$

Variable	Value
β_0	38.66645943863722
β_1	-3.335984211745958
PCR Efficiency	0.9941673919755294
$\bar{y}$	28.925
$\hat{x}_0$	831.9851413821814
s_p^2	0.00981666666666674
s_{xx}	14.787116116160096
$\bar{\bar{x}}$	3.4889132261647355
g	0.00031721429593360836
x_u (95% PI)	757.1503874590653 - 913.763660990488
$\bar{y}_u$ (95% PI)	28.78882848628346 - 29.061171513716534

Supplementary References

(1) Sun, S.; Meng, S.; Zhang, R.; Zhang, K.; Wang, L.; Li, J. Development of a New Duplex Real-Time Polymerase Chain Reaction Assay for Hepatitis B Viral DNA Detection. *Virol J* 2011, *8* (1), 227. https://doi.org/10.1186/1743-422x-8-227.

(2) Zhang, L.; Sun, Y.; Chang, L.; Jia, T.; Wang, G.; Zhang, R.; Zhang, K.; Li, J. A Novel Method to Produce Armored Double-Stranded DNA by Encapsulation of MS2 Viral Capsids. *Appl Microbiol Biot* 2015, *99* (17), 7047–7057. https://doi.org/10.1007/s00253-015-6664-4.

(3) Pizzamiglio, S.; Verderio, P.; Orlando, C.; Marubini, E. Confidence Interval for DNA/MRNA Concentration by Real-Time PCR. *Int J Biological Markers* 2007, *22* (3), 232–236. https://doi.org/10.1177/172460080702200312.

(4) Fieller, E. C. The Biological Standardization of Insulin. *Suppl J Royal Statistical Soc* 2018, *7* (1), 1–54. https://doi.org/10.2307/2983630.
